# High expression of B7-H6 in human glioma tissues promotes tumor progression

**DOI:** 10.18632/oncotarget.16391

**Published:** 2017-03-21

**Authors:** Tianwei Jiang, Wei Wu, Huasheng Zhang, Xiangsheng Zhang, Dingding Zhang, Qiang Wang, Lei Huang, Ye Wang, Chunhua Hang

**Affiliations:** ^1^ Department of Neurosurgery, Jinling Hospital, School of Medicine, Nanjing University, Nanjing, Jiangsu Province, China

**Keywords:** B7-H6, glioma, immunohistochemistry, RNAi, cancer progression

## Abstract

B7-H6, a new member of B7-family ligand, also known as NCR3LG1, plays an important role in NK cells mediated immune responses. Many studies have shown that it is highly expressed in various human cancers, and its expression levels are significantly associated with cancer patients’ clinicopathological parameters and postoperative prognoses. But, still the exact role of B7-H6 expression in human glioma remains elusive. In the present study, we have characterized the B7-H6 expression in the human glioma tissues as well as glioma cell lines, U87 and U251. We observed that B7-H6 was highly expressed in the human glioma tissues, and its expression was significantly associated with cancer progression. By using the RNA interference technology, we successfully ablated B7-H6 expression in human glioma cell lines to further study its contribution towards various biological features of this malignancy. Our study identified that the B7-H6 knockdown in U87 and U251 glioma cells significantly suppressed cell proliferation, migration, invasion, and enhanced apoptosis along with induction of cell cycle arrest. It thus suggested that B7-H6 play an important role in the regulation of the biological behavior of these glioma cells. However, the detailed mechanism of B7-H6 mediated regulation of glioma cancer cell transformation and its prognostic value merits further investigation.

## INTRODUCTION

Glioma, the most common tumor of the central nervous system, accounts for about 40–50% of the human adult brain tumors [1]. The patients with malignant glioma usually have a poor prognosis and a high rate of mortality [2]. Despite the availability of multiple strategies such as novel surgical treatments and effective radiation/chemotherapy for the diagnosis of high-grade glioma in recent decades, the overall 5-year survival rate of the patients still remains poor. Thus it is important to explore novel biomarkers that can not only benefit the early diagnosis of this malignancy but also help in deciding the targeted treatment strategy to improve patient's postoperative prognosis.

Natural killer (NK) cells are an important component of innate immune system and usually act as the first line of defense against numerous pathogens as well as some cancers [3]. The NK cells regulate both innate and adaptive immune responses via secreting pro-inflammatory cytokines and chemokines, mediating cytotoxic activity through direct lysis of the target cells [4]. These cell express activating receptors on their surface such as NKp30, NKp44 and NKp46, which contribute essentially in the recognition and elimination of abnormal target cells [5]. However, cancer cells escape NK-cell-mediated recognition in tumor microenvironment via expressing certain ligands like PD-L1 and so on [6, 7].

B7-H6, also known as NCR3LG1 or DKFZp686O24166, is a newly identified ligand in the B7 family [8]. It is a type I transmembrane protein, with considerable homology with B7-H1 and B7-H3 proteins [9]. It binds to the activating receptor NKp30 on natural killer cells, and initiates innate immune response for cellular transformation [8]. It has been demonstrated that the B7-H6 expression is not detected in normal human tissues, but was expressed by human tumor cells, and serve as a damage-associated molecular pattern to trigger innate immunity [8]. Pesce *et al*., has reported that reduced expression of NKp30 and the NK cells dysfunction in the peritoneal fluids of ovarian cancer patients, were significantly associated with the increased expression of soluble B7-H6 ligand in the peritoneal fluid [10]. A previous study has also reported that B7-H6 was highly expressed in human ovarian cancer tissues, and its expression level was significantly associated with cancer progression and patients’ prognoses [6].

In the present study, we have focused on analyzing the B7-H6 expression in human glioma tissues as well as human glioma cells lines, and to further understand its role in glioma progression using specific knockdown studies.

## RESULTS

### Analysis of B7-H6 expression in human glioma tissues and cancer cell lines

**Figure 1:**
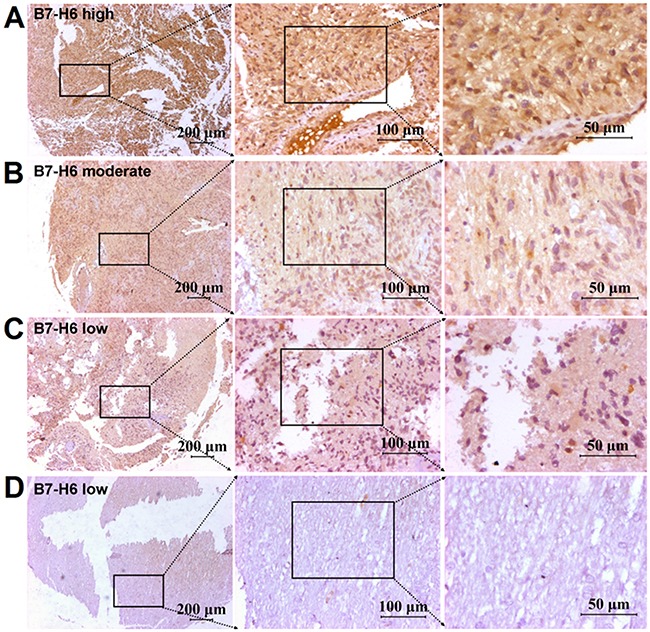
Immunohistochemical staining of B7-H6 in human glioma tissues B7-H6 expression detected by using immunohistochemical staining in human glioma tissues, and the positive B7-H6 staining could be found on the membrane and in the cytoplasm of cancer cells. **(A)** High B7-H6 expression in human glioma tissues. **(B)** Morderate B7-H6 expression in human glioma tissues. **(C)** Low B7-H6 expression in human glioma tissues. **(D)** Low B7-H6 expression in adjacent normal tissue. Scale bar = 200μm in left panel, scale bar = 100μm in middle panel, scale bar = 50μm in right panel.

**Figure 2:**
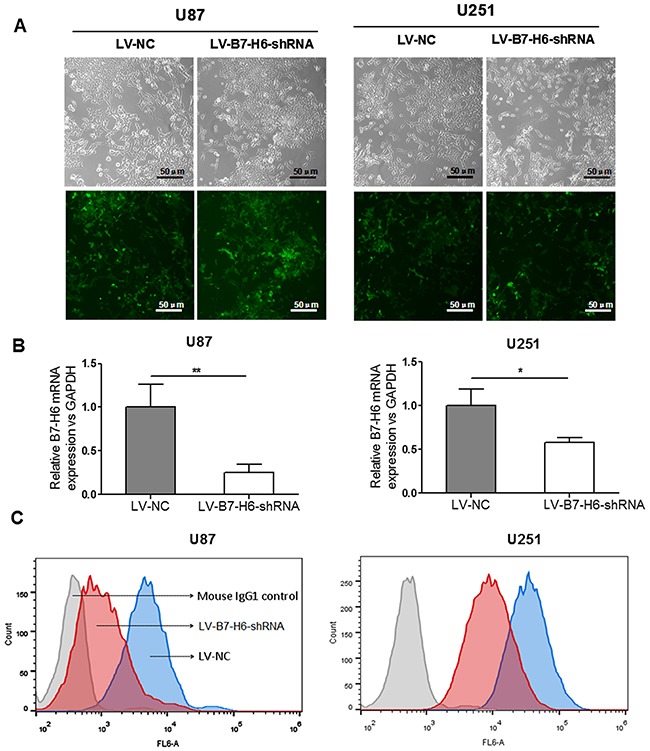
Confirmation of B7-H6 knockdown efficiency in human glioma cell lines **(A)** Confirmation of infection efficiency of glioma cell lines with recombinant lentivirus vector containing B7-H6 shRNA, through GFP expression analysis using fluorescence microscopy. **(B)** Validation of the B7-H6 mRNA expression after knock-down in U87 and U251 cells by using real-time RT-PCR analysis. **(C)** Validation of the B7-H6 mRNA expression after knock-down in U87 and U251 cells by using flow cytometry analysis.

The immunohistochemistry analysis showed positive staining for B7-H6, both on the membrane and in the cytoplasm of glioma cells to variant degrees Figure 1A, 1B, and 1C. Instead, normal control tissues displayed none or very weak staining for B7-H6, as shown in Figure 1D. Moreover, the flow cytometry analysis also showed B7-H6 expression on the membrane of the human cancer cell lines, U87 and U251, as shown in Figure 2C.

### Analysis of B7-H6 expression correlation with patient's clinical parameters

The correlation between patient's clinical parameters and glioma B7-H6 expression has been shown in Table 1. Our data suggested that B7-H6 expression in human glioma tissues was significantly associated with pathology type (*P*<0.001), grade (*P*=0.003), and tissue type (*P*=0.001). However, we did not find any correlation with patient's gender. Thus, these results indicated that higher B7-H6 protein expression on glioma tumor cells may be involved in the progression of this malignancy.

**Table 1:** Correlation of B7-H6 expression (H-score) with patients’ clinicopathological parameters

Clinicopathological Parameter	NO.	Score	Z/χ^2^	*P*
**Gender**				
Men	109	140(5-270)	0.921	0.357
Women	156	142.5(0-290)		
**Pathology type**				
Astrocytoma	171	140(0-285)	23.935	<0.001
Ependymoma	11	180(130-290)		
Glioblastoma	67	155(5-265)		
Cancer adjacent normal brain tissue	8	130(65-150)		
Normal brain tissue	11	100(10-130)		
**Grade**				
1-2	127	135(0-270)	3.008	0.003
3-4	58	160(5-290)		
**Group**				
Malignant	249	150(0-290)	13.908	0.001
Cancer adjacent normal brain tissue	8	130(65-150)		
Normal	11	100(10-130)		

### Validation of B7-H6 expression knockdown in glioma cell lines, U87 and U251

To investigate, if B7-H6 has any specific role in the biological regulation of glioma cells, we first knocked down its expression using recombinant lentiviral GFP vector containing, shRNA targeting B7-H6 gene (LV-B7-H6-shRNA virus) and the non-targeted shRNA sequence as control (LV-NC virus). The human glioma cell lines, U87 and U251 were infected with the lentiviral particles from these two vectors and were sorted using flow sorter (BD Aria II, USA) based on GFP expression. The efficiency of the infection was also confirmed by detecting GFP expression using fluorescence microscopy (Figure 2A). In parallel, we also validated knockdown efficiency by analyzing B7-H6 mRNA expression level with real-time RT-PCR analysis. Our data demonstrated that B7-H6 mRNA expression was significantly decreased in both cell lines, U87 and U251 cells infected with LV-B7-H6-shRNA in comparison to LV-NC control shRNA infection (*P*<0.01 and *P*<0.05, respectively, Figure 2B). In addition, the flow cytometry analysis also confirmed the reduced surface expression of B7-H6 protein on both glioma cell lines from LV-B7-H6-shRNA group, as shown in Figure 2C. Moreover, we also designed another siRNA to confirm the effect of down-expression of B7-H6 to the cellular function of glioma cell lines, and the B7-H6 mRNA expression level was also significantly decreased in the siRNA transient-transfection cells in contrast to the si-NC transient-transfection cells (Supplementary Figure 1).

### B7-H6 knockdown suppresses the proliferation and migration of human glioma cell lines

**Figure 3:**
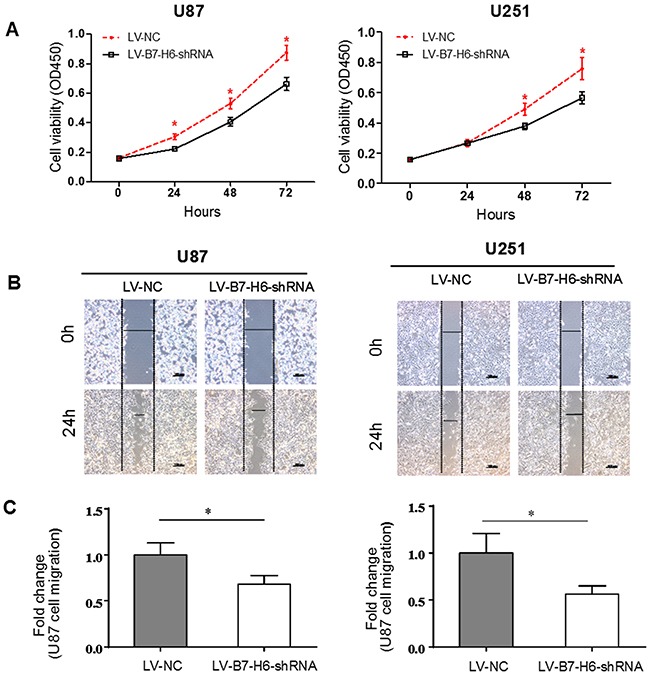
Effect of B7-H6 knockdown on glioma cells proliferation and migration **(A)** Analysis of cell proliferation in U87 and U251 cells in LV-NC and LV-B7-H6-shRNA groups by using CCK-8 assay. We examined the knockdown expression of B7-H6 on the cell proliferation rate *in vitro* by using CCK-8 assay in human glioma cell lines in both LV-B7-H6-shRNA and LV-NC groups. At 48 hours and 72 hours after seeding, the proliferation rate of LV-B7-H6-shRNA group cells was significantly lower than that of LV-NC group cells (*P*<0.05 respectively). **(B)** and **(C)**. The wound healing assay on the two human glioma cell lines in LV-B7-H6-shRNA group and LV-NC group showed that, the cell-free area of the LV-B7-H6-shRNA group was significantly wider than that of LV-NC group at 24 hours (both *P*<0.05) after drawing the scratch line on the monolayer cells.

Next to identify the specific role of B7-H6 in the growth of human glioma cells, we compared the cell proliferation rate of U87 and U251 cell lines infected with either LV-B7-H6-shRNA or LV-NC, using CCK-8 assay As shown in Figure 3A, we observed that after 24, 48 and 72 hrs, the proliferation rate of U87-LV-B7-H6-shRNA cells was significantly lower than that of U87-LV-NC (*P*<0.05), while U251-LV-B7-H6-shRNA cells showed significantly lower proliferation rate only after 48 and 72 hrs than U251-LV-NC (*P*<0.05). This data suggested that reduced B7-H6 expression on human glioma cells could suppress their cell proliferation. In addition, we also tested the role of B7-H6 in a wound healing assay, and as shown in Figure 3B, we observed that in both U87 and U251 cell lines, the cell-free area of the LV-B7-H6-shRNA group was significantly wider than that of LV-NC group, after 24 hrs (*P*<0.05, Figure 3C), suggesting that B7-H6 expression was also involved in the regulation of the migration ability of glioma cells. Moreover, the results from the siRNA method treated cells also confirmed that B7-H6 knockdown could significantly suppress the proliferation and migration of human glioma cell lines (Supplementary Figure 2).

### B7-H6 knockdown inhibits the invasive ability of glioma cancer cells

**Figure 4:**
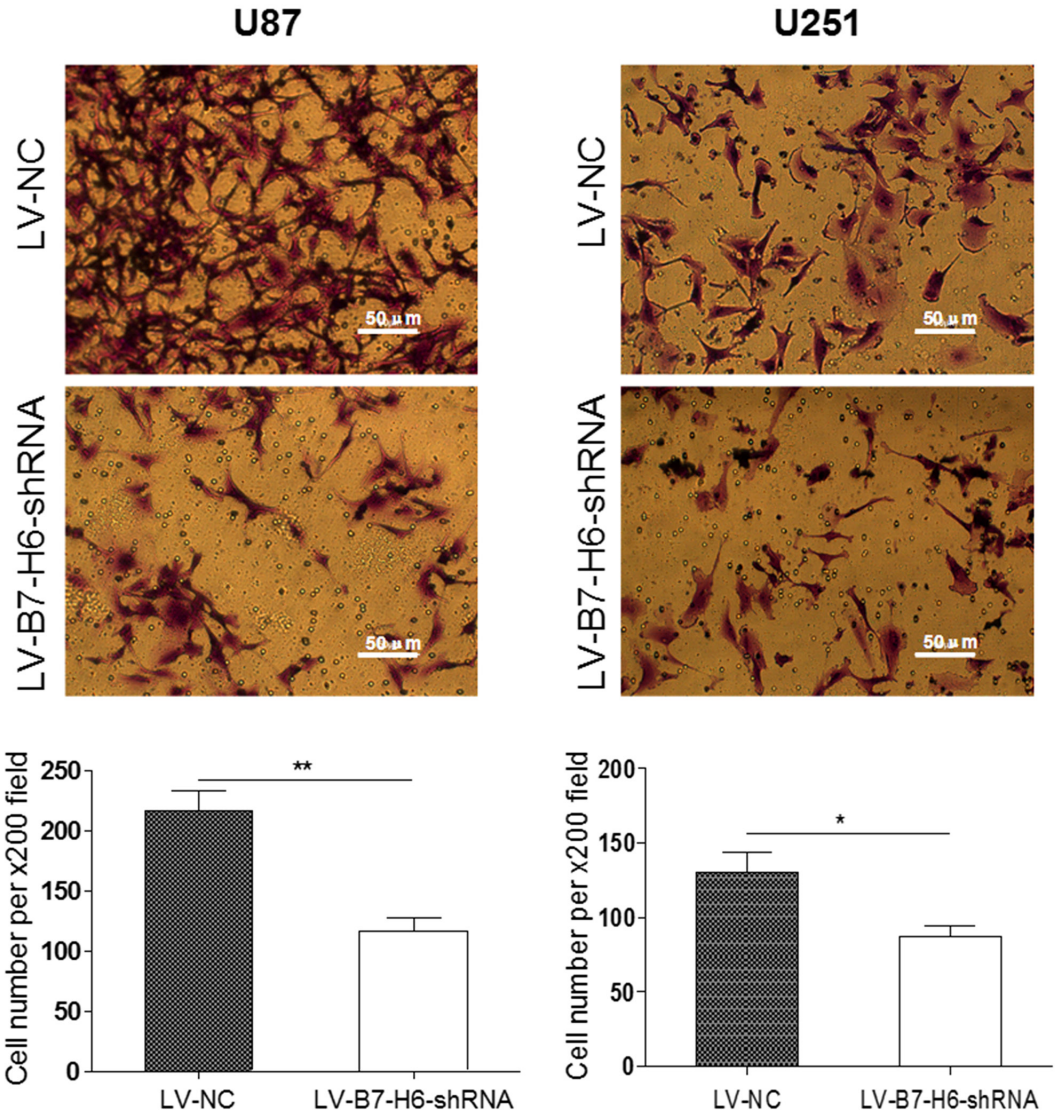
Effect of B7-H6 knockdown on the invasive ability of glioma cells The transwell invasion assay showed that the number of invaded cells stained with Cristal Violet was significantly less in the LV-B7-H6-shRNA group in contrast to the LV-NC group (U87: *P*<0.01, U251: *P*<0.05, respectively).

We further tested the contribution of B7-H6 expression in the glioma cell invasion, using transwell invasion assay. As shown in Figure 4, both U87 and U251 glioma cells from LV-B7-H6-shRNA group showed significantly reduced number of cells invading through the matrigel, as compared to cells from LV-NC group (*P*<0.01 and *P*<0.05, respectively). These results again suggested that B7-H6 expression in human glioma cells indeed play a role in the regulation of tumor invasion. Moreover, the results from the siRNA method treated cells also confirmed that B7-H6 knockdown could significantly inhibit the invasive ability of glioma cells (Supplementary Figure 3).

### B7-H6 knockdown enhances apoptosis and induces cell cycle arrest of human glioma cells

**Figure 5:**
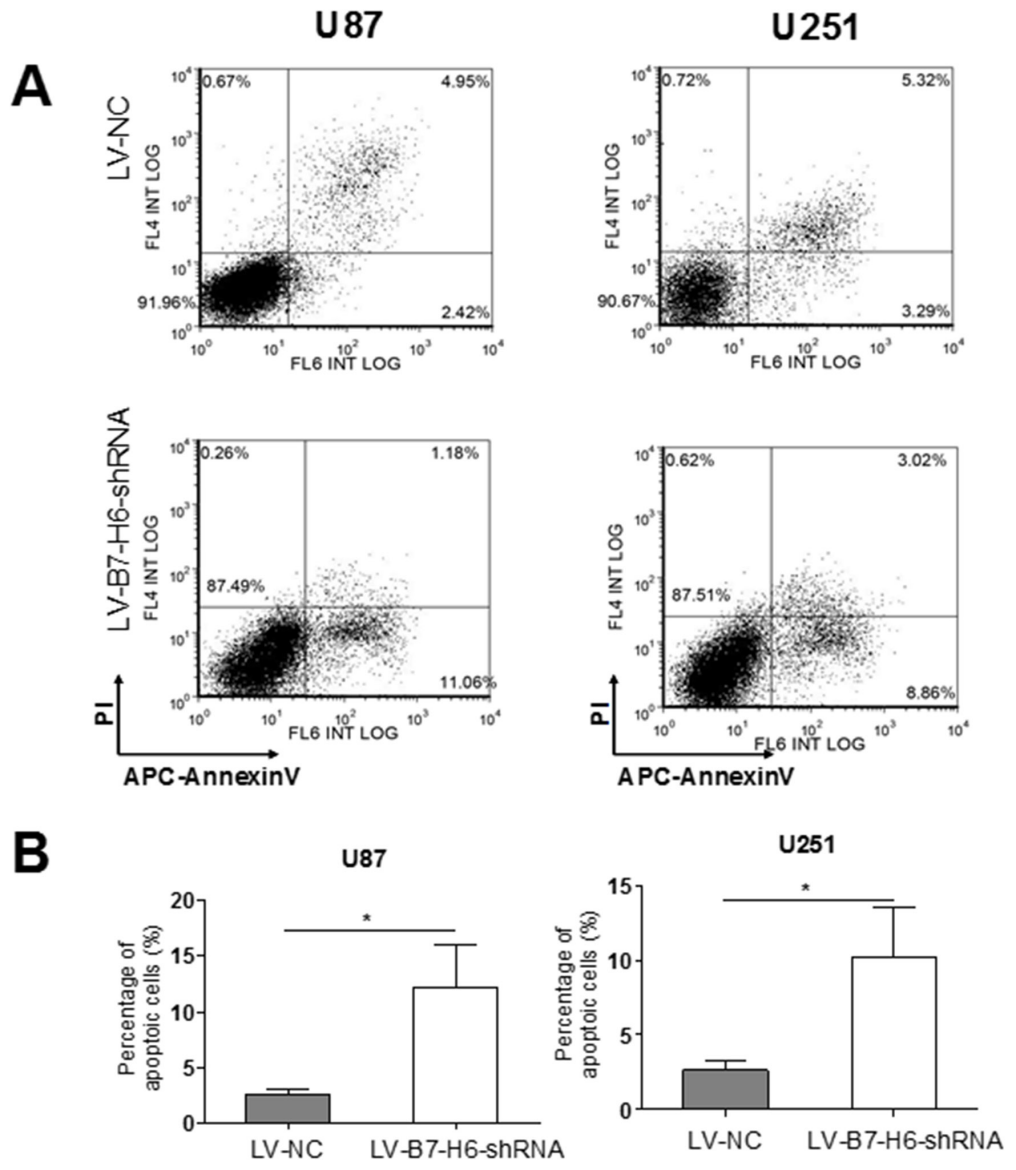
Effect of B7-H6 knockdown on the apoptosis of human glioma cells **(A)** The flow analysis showed that there were 11.06% apoptotic cells in U87-B7-H6-shRNA cells in contrast to 2.42% in U87-LV-NC cells, and 8.86% apoptotic cells in U251-LV-B7-H6-shRNA cells in contrast to 3.29% in U251-LV-NC cells. **(B)** The statistical analysis showed that the ratio of apoptotic cells was significantly less in LV-B7-H6-shRNA group in contrast to LV-NC group, both in U87 and U251 cells (*P*<0.05, respectively).

**Figure 6:**
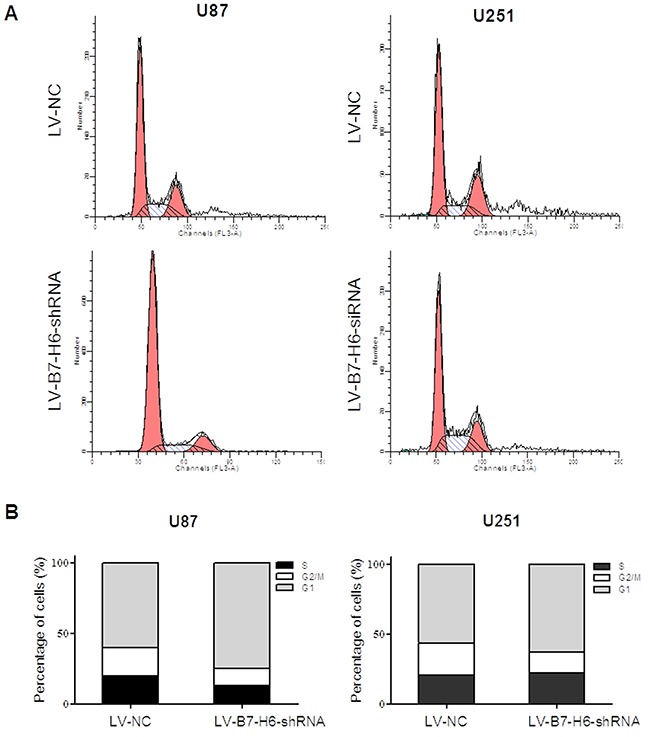
Effect of B7-H6 knockdown on the cell cycle regulation on human glioma cells The cell cycle analysis results showed that the cells from the LV-B7-H6-shRNA group, in both U87 and U251 cell lines, displayed increased percentage of cells in the G1-phase and decreased percentage in G2/M phases, in comparison to the cells from LV-NC group.

Finally, we tested the role of B7-H6 expression in glioma cell apoptosis and cell cycle regulation. As shown in Figure 5A and 5B, the cell death analysis using PI-AnnexinV staining revealed that down-regulation of B7-H6 expression in both U87 and U251 cell lines significantly increased the percentage of apoptotic cells in the LV-B7-H6-shRNA group as compared to LV-NC group (*P*<0.05). Furthermore, the cell cycle analysis results showed that, the cells from the LV-B7-H6-shRNA group, in both U87 and U251 cell lines, displayed increased percentage of cells in the G1-phase and decreased percentage in G2/M phases, in comparison to cells from LV-NC group (Figure 6). This data indicated that B7-H6 knockdown induced cell cycle arrest in glioma cells. Moreover, the results from the siRNA method treated cells also confirmed that B7-H6 knockdown could also lead to the increased percentage of cells in the G1-phase and decreased percentage in G2/M phases (Supplementary Figure 4).

## DISCUSSION

The B7 family of co-stimulatory molecules, also known as immune checkpoint molecules, have been shown to not only play an important role in mediating anti-tumor immune responses, but are over-expressed on cancer cell and have been implicated in human cancer progression. Some members of this family of molecules have also been considered as important immunotherapeutic targets against cancer, and have shown significant therapeutic effects [13]. The glioma is the most common primary brain tumor in adults but it still remains incurable with very poor prognosis and median survival of less than 15 months, due to the relapse and metastasis characteristics [14]. The immunotherapeutic approaches, such as vaccination, immune-check point blockade, adoptive T cell therapy, and even combination strategies, have shown some promising survival benefits for glioma patients [15].

It has been suggested that inhibition of NK cell mediated immunosurveillance, contributed importantly to the oncogenesis and cancer progression of human glioma [16]. The glioma cells express certain inhibitory molecules, which bind to the cell surface receptors on NK cells and results in the suppression of their function, and thus help themselves to escape immune response [17]. For instance, Bassoy*et al*. have demonstrated that blockade of inhibitory PD-1/B7H1 pathway promoted NK cells mediated killings of glioma stem cells, and thus suggested that PD-1 inhibition could be a feasible immune therapeutic approach against glioma [18]. Importantly, some other members of B7 family like 4IgB7-H3 and B7-H4 also has higher expression on tumor cells, and contributes to immune evasion by engaging with inhibitory receptors on NK cells. But, B7-H6, a new member of the B7 family, binds to the activating receptor NKp30 on NK cells, and promotes the innate immune response [8]. Thus it seems to have the opposite function in regards to immune response regulation as compared to other members of B7 family. But still, a study by Semeraro *et al*. showed elevated soluble B7-H6 levels contained in sera may inhibit NK functions *in vitro* and correlated with down-regulation of NK-p30 on NK cells, which leaves its exact effect controversial [19]. In our study, we did not detect B7-H6 mRNA expression in normal tissues or cancer tissues of the brain, but we observed significantly higher expression of B7-H6 protein on tumor tissues in contrast to the normal tissues, and the higher expression level of B7-H6 was significantly associated with cancer progression and the pathological type. However, B7-H6 mRNA expression data from TCGA in glioma patients showed that the subgroup with low B7-H6 mRNA expression showed poorer survival than the subgroup with high B7-H6 expression (Supplementary Figure 5, *P*=0.028), which suggests the potential post-transcriptional regulation of B7-H6 in glioma progression. It has been proposed that up-regulation of B7-H6 expression on tumor cells is probably stress-induced and help in self-recognition by NK cells [20]. Further, Wu *et al*. have also reported that B7-H6-specific chimeric antigen receptors lead to the tumor elimination and enhanced host antitumor immunity [21].

However, there have been some additional linking B7-H6 expression with cancer progression and suggest it to be an important therapeutic target, indicating the involvement of different mechanism than immune cell regulation. Zhou *et al*. reported that B7-H6 was over-expressed in ovarian cancer tissues, and the expression level was significantly associated with cancer progression and patient's prognosis [6]. Another study by Fiegler *et al*. reported that down-regulation of B7-H6 was associated with decreased B7-H6 reporter activity and reduced histone acetylation at the B7-H6 promoter, suggesting the novel immunotherapeutic strategy of combining B7-H6 targeting with histone deacetylase inhibitors [8]. Wu *et al*. also reported that B7-H6-specific chimeric antigen receptors could lead to the tumor elimination and enhance the host anti-tumor immunity [21]. The study by Schlecker*et al*., demonstrated that tumor cells release B7-H6, the ligand for the activating NK-cell receptor NKp30, by ectodomain shedding, resulting in reduced surface level of its expression on tumor cells and subsequently reduced NKp30-mediated recognition by NK cells, and thus helping tumor cells escape NK cell mediated killing [6]. Our study demonstrated that higher B7-H6 expression in tumor tissues was significantly associated with pathological type and tumor grade, thus suggesting that B7-H6 was involved in the physio-pathological progression of human glioma as the result also indicated in a recent study by Guo *et al*. [22].

As alluded before, many members from B7 family ligands not only contributed to the regulation of cell mediated immune response, but have also been implicated in regulating the biological behaviors of cancer cells that eventually leads to cancer progression and metastasis [23]. For instance, it has been demonstrated that the B7 family co-inhibitory members B7-H1 and B7-H3 have aberrant expression on human cancer cells, and play an important role in the regulation of biological behavior of cancer cells through different mechanism independent of immune cell regulation [24]. A recent study showed that B7-H6 knockdown significantly inhibited the tumor progression and enhanced the chemo-sensitivity in B-cell non-Hodgkin lymphoma [25]. Consistent with this, we in our study also observed that the knockdown of B7-H6 in glioma cell lines significantly suppressed cell proliferation, migration, invasion, and enhanced apoptosis along with induction of cell cycle arrest. All these observations, suggested that B7-H6 play an important role in the regulation of the biological behaviors of glioma cells.

## MATERIALS AND METHODS

### Patients and tissues samples

The glioma tissue array, GL2083a was purchased from Alenabio Co., Ltd. (Shanxi Xi’an, P.R. China), while another array, HBra-Gli060PG was purchased from Shanghai Outdo Biotech Co., Ltd. (Shanghai, P.R. China). From these arrays, the data from 249 cases of glioma tissues and 19 cases of normal control tissues was included in the final analysis. The confirmation of the tumor tissues as glioma was performed using hematoxylin and eosin (H&E) staining after surgical resection. The detailed clinical parameters of the patients are shown in Table 1. The protocols for the current study were approved by the ethics committee of the Jinling Hospital.

### Antibodies and major reagents

Rabbit anti-human B7-H6 polyclonal antibody was purchased from Abcam (Cambridge, MA, USA), and APC labeled mouse anti-human B7-H6 monoclonal antibody was purchased from R&D Systems (Minneapolis, MN, USA). APC-conjugated mouse IgG1 Isotype control was purchased from eBioscience (San Diego, CA, USA). The HRP-labeled goat anti mouse/rabbit secondary antibody (K500711, ready to use) was purchased from Dako (Glostrup, Denmark). The RNeasy Mini Kit was purchased from Qiagen (Valencia, CA, USA), and SYBR Green Master Mix kits were purchased from TaKaRa (Dalian, China). DMEM and fetal bovine serum (FBS) were purchased from Gibco (Cambrex, MD, USA).

### Cell lines and cell culture

Human glioma cell lines, U251 and U87 obtained from Chinese Academy of Sciences, Shanghai Institutes for Biological Sciences, were cultured in DMEM, supplemented with 10% FBS, and incubated at standard culture conditions (5% CO_2_, 37°C).

### Immunohistochemistry

The glioma tissue array blocks were cut into 4-μm-thick sections, and were dewaxed in xylene, rehydrated and graded in ethanol solutions. Antigen was retrieved by heating the tissue sections at 100°C for 30 min in EDTA (1mmol/L, pH9.0) solution, and the sections were later immersed in a 0.3% hydrogen peroxide solution for 30 min to block endogenous peroxidase activity. After rinsing with phosphate buffered saline (PBS) for 5 min, sections were then blocked with 3% BSA at room temperature for 30 min, and later incubated with purified rabbit anti-human B7-H6 antibody (1:150 dilution) at 4 °C overnight. The negative control did not have the primary antibody. Next, the sections were incubated with HRP-labeled goat anti rabbit secondary antibody, according to the manufacture's instruction, and followed by incubation with diaminobenzene as the chromogen, and hematoxylin as the nuclear counterstain. Finally, the sections were dehydrated, cleared and mounted.

### Evaluation of immunohistochemical staining

All slides were examined independently by two senior pathologists who were not informed of the patients’ clinical parameters. The B7-H6 immunostaining densities were assessed according to the *H-score* method which has been described in the previous publication [11]. Briefly, H-score was calculated as follows = (% tumor cells unstained x0) + (% tumor cells stained weak x1) + (% tumor cells stained moderate x2) + (% tumor cells stained strong x3), and it ranged from 0 (100% negative tumor cells) to 300 (100% strong staining tumor cells). Results obtained from the five areas of the same section by the two pathologists were averaged and statistically analyzed for staining density.

### B7-H6 RNAi lentivirus generation, infection and cell sorting

The lenti-viral vector system was purchased from Clontech Laboratories Inc. (Mountain View, CA, USA). This vector system includes three plasmids: the pLVX-U6-GFP-puro vector, psPAX vector and pMD2G vector. The small hairpin RNA against the human gene B7-H6 (NM_001202439.2; GenBank) was subcloned into the pLVX-U6-GFP-puro vector, which contained U6 promoter, GFP gene and puromycin gene (puro). The targeting siRNA sequence was 5’-CATCAAGAATATGGATGGCACATTT-3’, while the non-targeted control sequence was 5’-TTCTCCCCGAACAACAACGTGTCACCACCACGT-3’. The recombinant lentivirus shRNA targeting B7-H6 (LV-B7-H6-shRNA virus) as well as the non-targeted control lentivirus (LV-NC virus) were produced by transient transfection of HEK293 cells. All virus stocks were produced by lipofectamine-mediated transfection. After 48 h of post-transfection, cell supernatants containing viral particles were filtered using the 0.45-μm Steriflip vacuum filtration system (Millipore, MA, USA) and concentrated by ultracentrifugation at 25000 rpm at 4˚C. The titer of the virus was tested according to the expression level of GFP. The day before infection, the U87 and U251 cells were seeded on dishes with a confluence of 30-40%. On the day of infection, the cells were infected by packaged lentiviral production. And subsequently propagated in selection medium containing puromycin (2 mg/ml) for at least 1 week. Finally, the infected cells from LV-B7-H6-shRNA group and LV-NC group, were analyzed by flow cytometry (Canto II, BD, USA) and positive infected cells were sorted based on the GFP signal (Aria II, BD, USA).

### B7-H6 RNAi lentivirus generation, infection and cell sorting

The human glioma cell lines, U87 and U251 were used for B7-H6 RNAi knockdown studies. The small hairpin RNA against the human gene B7-H6 (NM_001202439.2; GenBank) was cloned in the lentiviral gene transfer vector encoding green fluorescent protein (GFP). The targeting siRNA sequence was 5’-CATCAAGAATATGGATGGCACATTT-3’, while the non-targeted control sequence was 5’-TTCTCCCCGAACAACAACGTGTCACCACCACGT-3’. The recombinant lentivirus shRNA targeting B7-H6 (LV-B7-H6-shRNA virus) and the non-targeted control lentivirus (LV-NC virus) were prepared and the U87 and U251 cell lines were infected. The infected cells from both cell lines were categorized into LV-B7-H6-shRNA group and LV-NC group, based on the shRNA infection. Finally, the infected cells were analyzed by flow cytometry (Canto II, BD, USA) and positive infected cells were sorted based on the GFP signal (Aria II, BD, USA).

### Real-time reverse transcriptase-polymerase chain reaction

Real-time reverse transcriptase-polymerase chain reaction (RT-PCR) was performed to confirm the knockdown of B7-H6 mRNA expression. Total RNA from U87 and U251 cell lines was extracted using TRIzol (Invitrogen) reagent, and was then reverse transcribed into cDNA by using RT reaction kit (Promega). Real-time PCR was performed by using the ABI 7600 system (Applied Biosystems, USA) according to the manufacturer's instruction and SYBR Green was used as a DNA-specific fluorescent dye. Primer sequences for detection of the reference gene, GAPDH and the target gene B7-H6 were synthesized as follows, the human GAPDH, forward primer: 5’-TGACTTCAACAGCGACACCCA, and the reverse primer: 5’-CACCCTGTTGCTGTAGCCAAA-3’; the human B7-H6 forward primer: 5’-CTCCTGATT CTGCTGTGGGC-3’, and reverse primer: 5’-GTCGG AATGCCTCTTGGTGA-3’. The RT-PCR products for B7-H6 and GAPDH genes were also confirmed by using electrophoresis on 1.8% agarose gel containing 0.1% ethidium bromide. Images of the fluorescent bands were captured using Bio-Rad gel documentation system.

### Cell proliferation analysis

The cell proliferation was evaluated by Cell Counting Kit-8 (CCK-8, Beyotime, Shanghai, China), according to the manufacturer's instruction. Briefly, the LV-B7-H6-shRNA and the LV-NC groups of U87 and U251 cells (5×10^4^, respectively) were seeded into each separate well of a 96-well plate and cultured in 100μl of DMEM supplemented with 10% FBS. At the indicated time points, medium was replaced with CCK-8 reagent (10 μl CCK-8 and 90 μl DMEM), and the cells were further incubated for 1 h. Absorbance was measured for each well at a wavelength of 450 nm. Relative increase or decrease in the absorbance values at 450 nm in the experimental wells relative to the control wells, indicated cell growth or death, respectively. Cell growth was monitored every 24 hrs over 3 days. All experiments were independently repeated at least three times.

### Wound healing assay

Cell migration was evaluated by the wound scrape assay to determine if B7-H6 has some role in the regulation of migration ability of glioma tumor cells. Briefly, the cells from the LV-B7-H6-shRNA and the LV-NC group were incubated in 6-well plates. A small wound was made in the 90% confluent monolayer by using a 200 μl pipette tip in a lengthwise stripe. Cells were then washed twice with PBS and further incubated in serum-free DMEM medium at 37°C in a 5% CO_2_ incubator for 24 hours. Photographs were then taken at the indicated time points, and the wound width was measured at 100X magnification by using a BX50 microscope (Olympus^®^) with a calibrated eyepiece grid (1 mm / 20 μm graduation). Ten measurements were made at random intervals along the wound length and the data were averaged to express as a percent of the original width. The experiment was done in triplicate.

### Transwell invasion assay

The transwell culture system was used to evaluate the invasive ability of U87 and U251 cells ablated for B7-H6, as described in the previous study [12]. In brief, the upper portion of Transwell^®^ inserts with a 8 μm pore size and 6.5 mm diameter was coated with 20 μl matrigel diluted 1:3 in serum-free DMEM and incubated at 37 °C for 4 hours. In addition, LV-B7-H6-shRNA and LV-NC group cells were harvested by trypsinization after 12 hours of serum starvation, and were suspended in a serum-free DMEM medium at a dilution of 1×10^6^/ml. The 200 μl of the cell suspension was added to the upper chamber of coated inserts placed in the wells of a 24-well plate with 600 μl DMEM medium containing 10% FBS in the bottom chamber as chemo attractant. After incubation at 37 °C for 24 hours in a 5% CO_2_ atmosphere the non-invading cells and matrigel were removed from the upper chamber with cotton tipped swabs. The insert were rinsed with PBS and cells on the filters were fixed with methanol for 30 minutes and stained with crystal violet solution (Sigma). The number of invading cells on the filters was counted in 5 random fields per filter at 100X magnification in triplicate wells of each group.

### Apoptosis assay

Apoptosis-mediated cell death was examined using APC-Annexin V apoptosis detection kit (BD Biosciences, San Diego, CA, USA) according to the manufacturer's instructions. Briefly, the U87 and U251 cells from LV-B7-H6-shRNA and LV-NC groups were harvested. 1×10^6^ cells from each group were washed with phosphate-buffered saline (PBS) and resuspended in 100 μl binding buffer, and then incubated with 5 μl of APC-Annexin V and 5 μl of propodium iodide (PI) solutions. After 15 min incubation in the dark, the stained cells were analyzed by flow cytometry (Beckman Coulter, Epics XL) using the FlowJo 10.0.6.software.

### Cell cycle analysis

The LV-B7-H6-shRNA and the LV-NC cells (1×10^6^, respectively) were first washed with PBS and then fixed with 70% ice-cold ethanol at 4°C for overnight. After washing twice with PBS, cells were then stained with a solution containing 200 μg/ml of propidium iodide, 0.1% sodium azide, 0.1% Triton-X100 and 10 μg/ml of RNAses for 2~4 hrs in the dark at room temperature. The stained cells were analyzed for subG1, S and G2 peaks by flow cytometry (Beckman Coulter, Epics XL) using the Modfit software.

### Statistical analyses

Statistical analyses were performed by using the GraphPad Prism 5.0 software package (GraphPad Software, Inc., San Diego, USA). The paired student's *t*-test, Wilcoxon rank test, Chi-square test or the survival analysis were appropriately used. The P-value of <0.05 represented the significance.

## SUPPLEMENTARY MATERIALS FIGURES


