# Supplementary material for: High expression of B7-H6 in human glioma tissues promotes tumor progression
Source: Oncotarget. 2017 Mar 21;8(23):37435–47. doi: 10.18632/oncotarget.16391 (PMC5514920; doi:10.18632/oncotarget.16391)
Supplement: Supplementary file 1 [file oncotarget-08-37435-s001.pdf]

High expression of B7-H6 in human glioma tissues promotes tumor progression

MATERIALS AND METHODS

siRNA knockdown of B7-H6 in U87 and U251 cells

U87 and U251 cells were plated onto 6-well plates and allowed to grow to sub-confluent. Cells were transiently transfected with negative control siRNA (5'-CCGAGG UUCCUUAUGUGCAAGCCUU-3') or B7-H6 siRNA (5'- CCGAGGUUCCUUAUGUGCAAGCCUU-3') by lipofectamine RNAi MAX reagent (Invitrogen, America) in OPTI-MEM medium (Gibco, America) for 6 h, and then incubated in DMEM with 10% FBS. Cells were prepared to be used for further experiments.

Statistical analyses

All statistical analyses were performed by using GraphPad Prism 5.0 software package (GraphPad Software, Inc., San Diego, USA). Cut-off Finder (http://molpath.charite.de/cutoff) was used to determine a cut-off point and stratify patients into two groups. Kaplan-Meier method and the log-rank test were used for comparing survival curves. The *P* value of less than 0.05 based on the two-sided test was considered to be statistically significant.

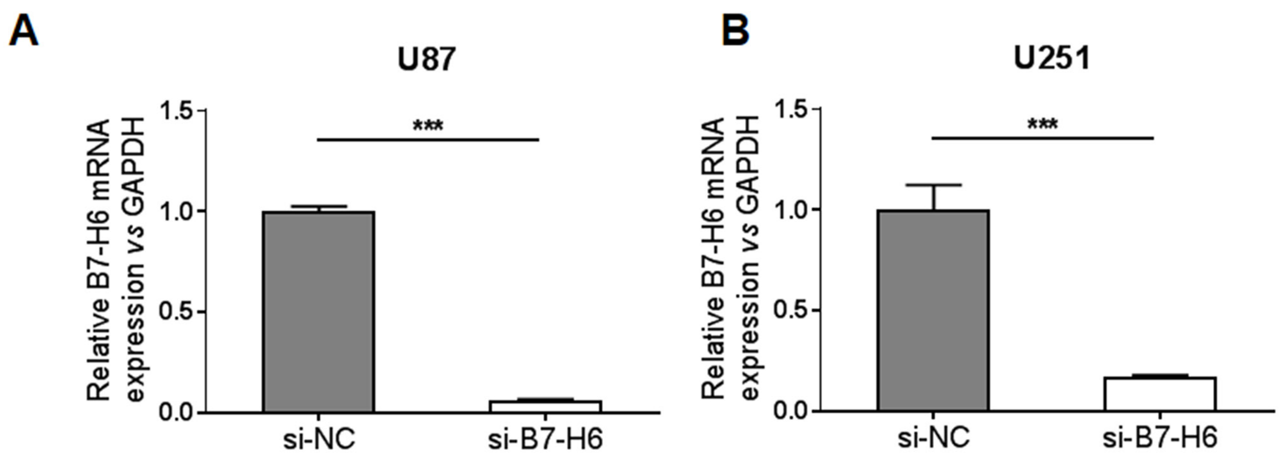

Supplementary Figure 1: Confirmation of B7-H6 knockdown efficiency in human glioma cell lines. **(A)** Validation of the B7-H6 mRNA expression level after knock-down in U87 cells by using real-time RT-PCR analysis ($P<0.001$). **(B)** Validation of the B7-H6 mRNA expression level after knock-down in U251 cells by using real-time RT-PCR analysis ($P<0.001$).

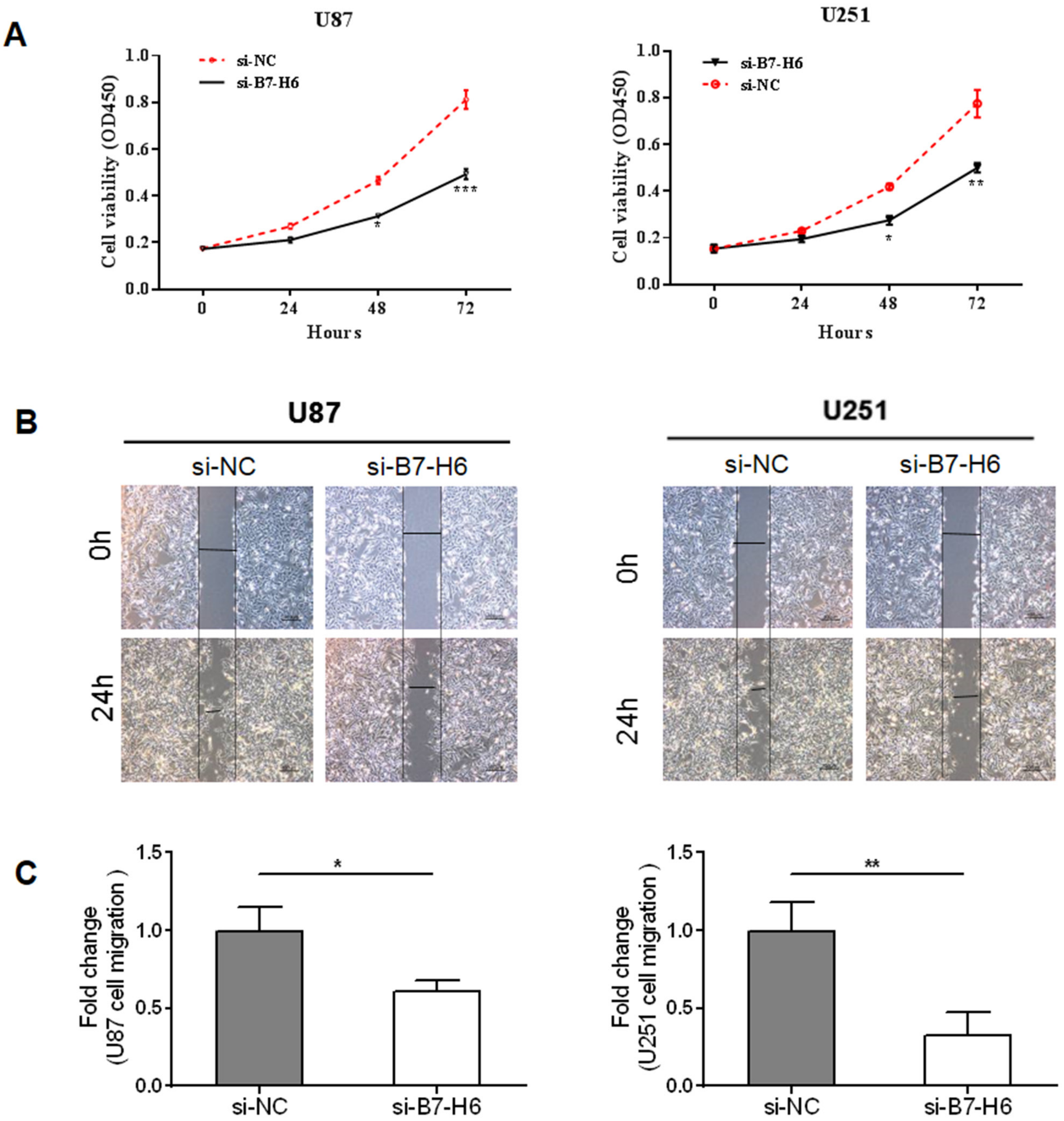

Supplementary Figure 2: Effect of B7-H6 knockdown on glioma cells proliferation and migration. **(A)** Analysis of cell proliferation in U87 and U251 cells in si-NC and si-B7-H6 groups by using CCK-8 assay. We examined the knockdown expression of B7-H6 on the cell proliferation rate *in vitro* by using CCK-8 assay in human glioma cell lines in both si-B7-H6 and si-NC groups. At 48 hours and 72 hours after seeding, the proliferation rate of si-B7-H6 group cells was significantly lower than that of si-NC group cells ($P<0.05$ and $P<0.001$ for U87, $P<0.05$ and $P<0.01$ for U251, respectively). **(B)** and **(C)**. The wound healing assay on the two human glioma cell lines in si-B7-H6 group and si-NC group showed that, the cell-free area of the si-B7-H6 group was significantly wider than that of si-NC group at 24 hours ($P<0.05$ and $P<0.01$ respectively) after drawing the scratch line on the monolayer cells.

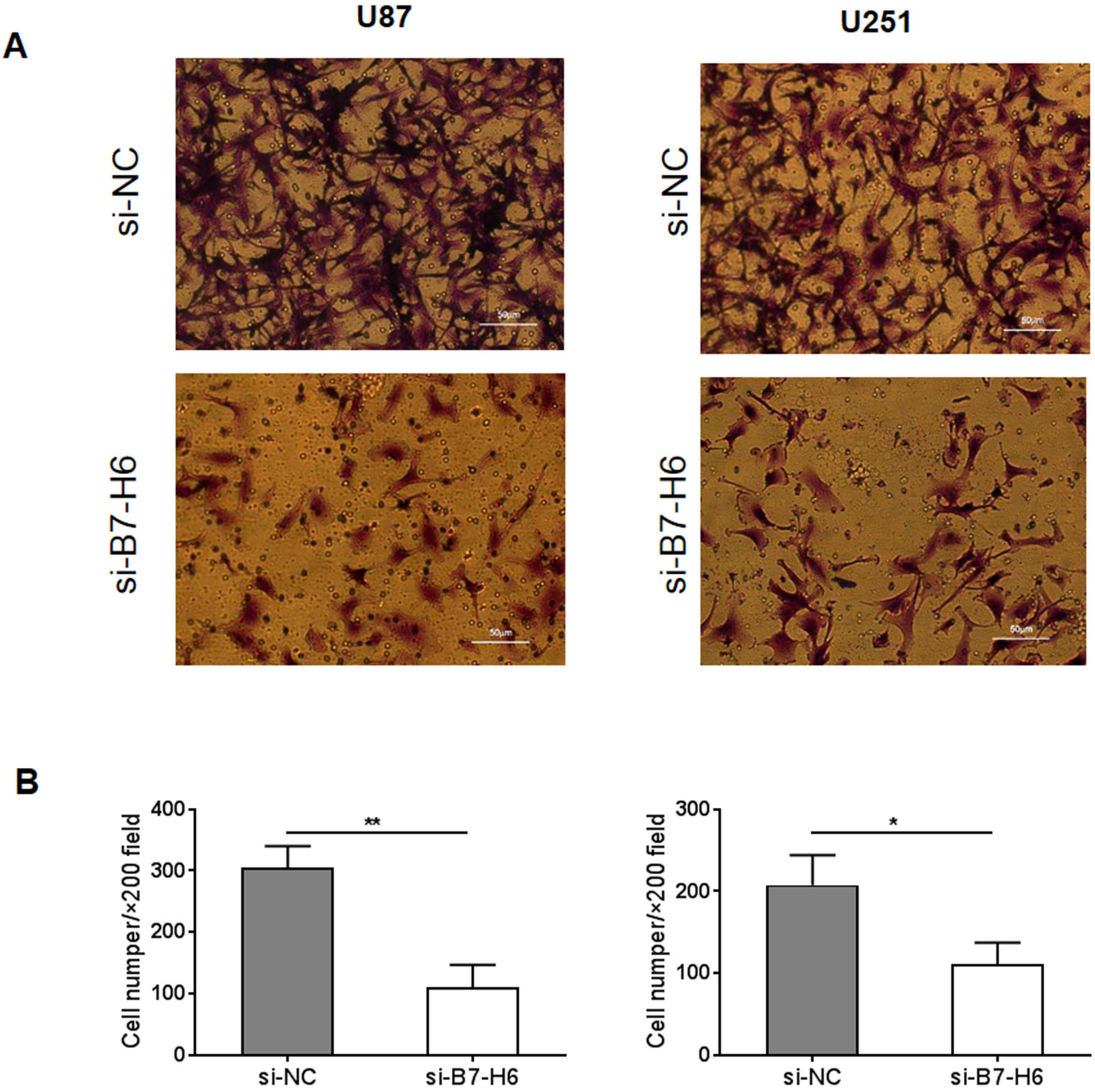

Supplementary Figure 3: Effect of B7-H6 knockdown on the invasive ability of glioma cells. The transwell invasion assay showed that the number of invaded cells stained with Cristal Violet was significantly less in the si-B7-H6 group in contrast to the si-NC group (U87: $P<0.01$, U251: $P<0.05$, respectively).

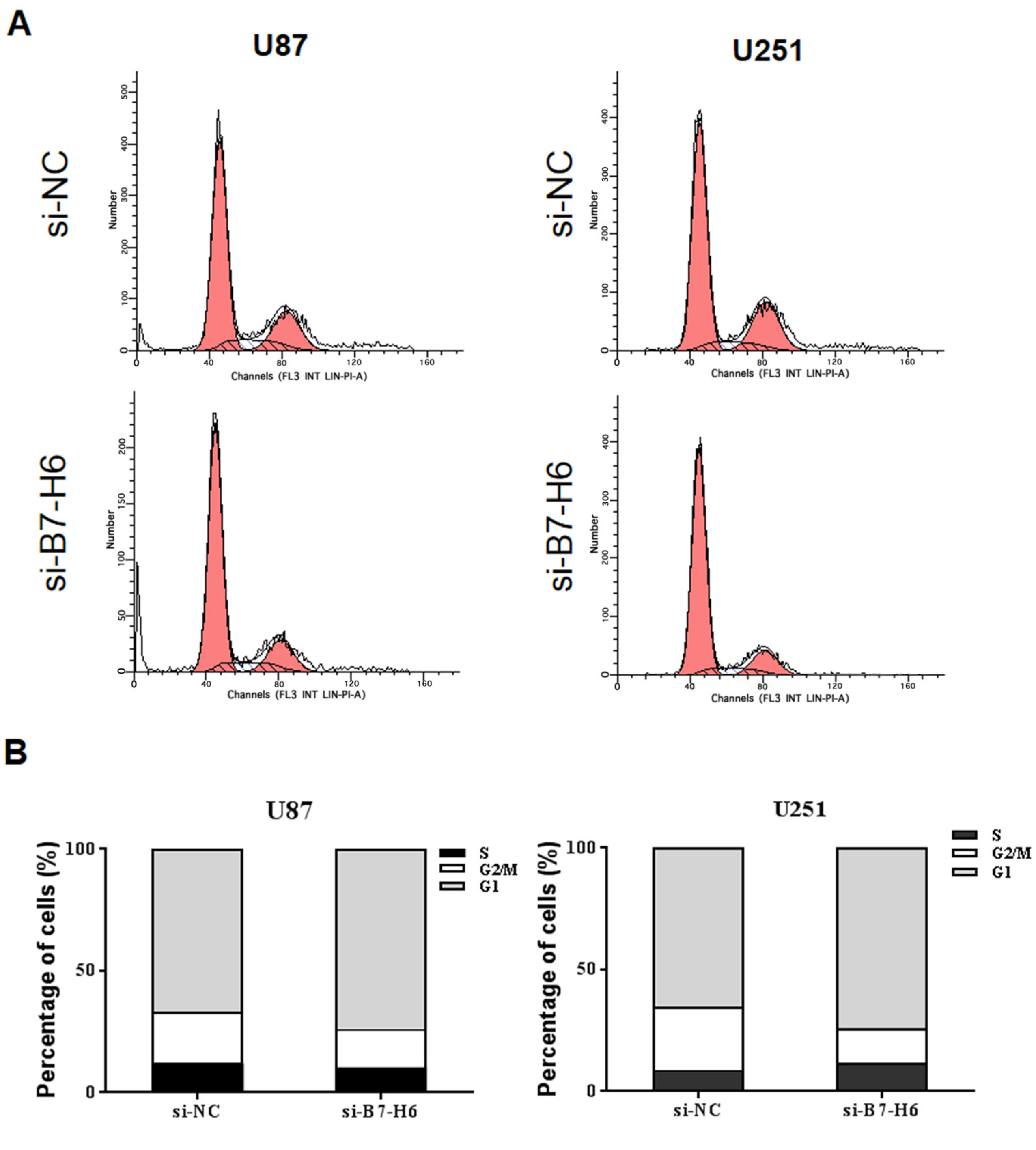

Supplementary Figure 4: Effect of B7-H6 knockdown on the cell cycle regulation on human glioma cells. The cell cycle analysis results showed that the cells from the si-B7-H6 group, in both U87 and U251 cell lines, displayed increased percentage of cells in the G1-phase and decreased percentage in G2/M phases, in comparison to the cells from si-NC group.

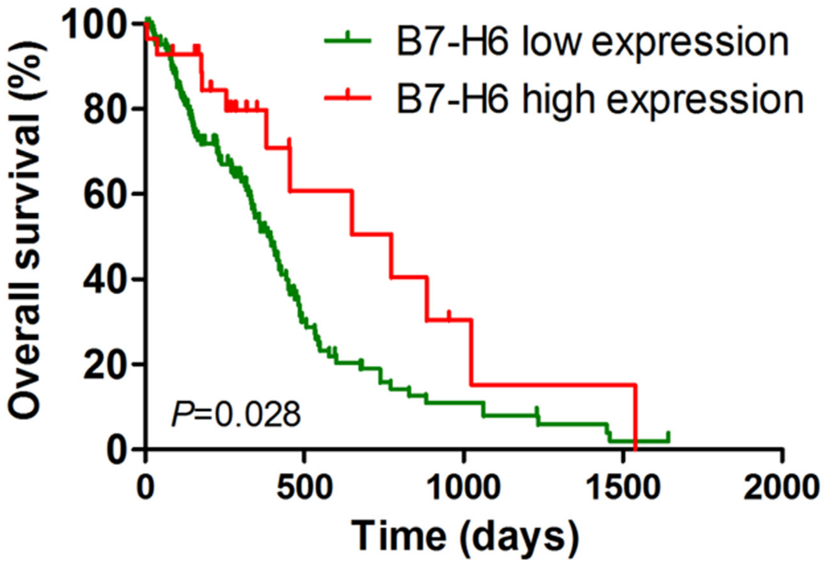

Supplementary Figure 5: B7-H6 mRNA expression data from TCGA in glioma patients. B7-H6 mRNA expression data from TCGA in glioma patients showed that the subgroup with low B7-H6 mRNA expression showed poorer survival than the subgroup with high B7-H6 expression (*P*=0.028).
